# Editorial: Advancing our understanding of the impact of dynamics at different spatiotemporal scales and structure on brain synchronous activity, volume II

**DOI:** 10.3389/fncom.2024.1386652

**Published:** 2024-03-05

**Authors:** Thanos Manos, Chris G. Antonopoulos, Antonio M. Batista, Kelly C. Iarosz

**Affiliations:** ^1^ETIS Lab, ENSEA, CNRS, UMR8051, CY Cergy-Paris University, Cergy, France; ^2^School of Mathematics, Statistics and Actuarial Science, University of Essex, Wivenhoe Park, United Kingdom; ^3^Department of Mathematics and Statistics, State University of Ponta Grossa, Ponta Grossa, Brazil; ^4^Science Graduated Program, State University of Ponta Grossa, Ponta Grossa, Brazil; ^5^Institute of Physics, University of São Paulo, São Paulo, Brazil; ^6^Exact Sciences, Natural and Engineering, University Center, Telêmaco Borba, Brazil

**Keywords:** brain dynamics, neural networks, plasticity, synchronization, collective activity, neuron models

The study of complex networks in neuroscience, coupled with dynamical models, has emerged as a powerful approach to unraveling the intricate workings of the brain across species. By representing neural systems as networks of interconnected nodes, researchers can gain insights into the brain's organizational principles, information processing mechanisms, and emergent dynamics (see e.g., Bullmore and Sporns, 2009; Manzoni et al., 2020). Synaptic adaptation, a fundamental aspect of neural plasticity, plays a crucial role in shaping network dynamics and underpins various cognitive processes, including learning and memory (see e.g., Ooyen and Butz-Ostendorf, 2017). Understanding how synaptic connections evolve over time in response to activity patterns is essential for elucidating the mechanisms underlying brain function and dysfunction. Furthermore, the integration of stimulation techniques, such as optogenetics, deep brain stimulation or transcranial magnetic stimulation, provides a means to modulate network activity with high spatiotemporal precision (see e.g., Acharya et al., 2022).

Leveraging complex network analysis and dynamical modeling, researchers can investigate how alterations in brain network organization are related to neurological disorders (see e.g., Stam, 2014). Moreover, understanding the mechanisms by which synaptic adaptation influences network dynamics can provide insights into disease pathology and potential therapeutic targets (see e.g., Berner et al., 2023; Sawicki et al., 2023). Translational studies involving both human subjects and animal models, such as mice, facilitate the validation of findings across species and provide valuable insights into the generalizability of therapeutic interventions. Thus, the integration of complex networks, dynamical models, synaptic adaptation, and stimulation techniques represents an approach with significant potential for advancing our understanding of brain function and informing novel therapeutic strategies for neurological and psychiatric disorders.

This Research Topic aims to enhance comprehension of the influence of dynamics across various spatiotemporal scales, as well as the roles of structure and plasticity (neural connectivity properties in general) in shaping synchronous brain activity. To achieve this objective, it serves as a platform for interdisciplinary investigations drawing upon analytical and computational approaches from diverse fields, including complex systems, biophysics, and computational neuroscience.

Spontaneous low-frequency oscillations are integral to brain function, yet their origin remains elusive. In Li et al., the authors followed a hybrid approach utilizing optical imaging and electrophysiological recordings were employed to examine these oscillations in awake and anesthetized mice following N omega-nitro-L-arginine methyl ester (L-NAME) administration. The analysis revealed a significant alteration in the spectrum of local field potential (LFP) signals induced by L-NAME, accompanied by heightened energy and spatial synchronization. This observation suggests a disparity in the underlying mechanisms governing these oscillations, highlighting the complexity of their origin. The proposed neurovascular model offers insights into the multifaceted nature of spontaneous low-frequency oscillations, suggesting their emergence from diverse sources within the brain.

In Oprisan et al., the authors explored the effects of cocaine administration and parvalbumin-type interneuron stimulation on local field potentials (LFPs) within the medial prefrontal cortex (mPFC) using optogenetic tools in six mice. The adaptive Empirical Mode Decomposition (EMD) method was utilized to decompose LFP signals into seven orthogonal Intrinsic Mode Functions (IMFs), aligning with known brain activity frequencies. The power density distribution followed a power law with an average scaling exponent of ~1.4 across IMF frequencies (2–2,000 Hz). Their findings suggest that the scaling exponent for cocaine appears marginally reduced compared to the control condition, indicating that neural activity avalanches induced by cocaine exhibit extended durations and larger magnitudes.

Simplicial complexes, mathematical structures capturing higher-order interactions in networks, gain importance in neuronal systems due to biological context and past findings. The study conducted by Mehrabbeik et al. delves into a higher-order network employing the memristive Rulkov model, using master stability functions to assess its synchronization properties. Higher-order connections turned out to alter synchronization patterns, particularly reducing multi-node chemical coupling. These findings highlight how enlarging network size can bolster synchronization by reducing coupling parameter values. A cluster synchronization arose at elevated electrical coupling strengths, suggesting a departure from complete synchronization among neurons.

The dynamic equilibrium of excitatory and inhibitory balance across various brain regions is crucial for modulating neural input/output relationships within cortical networks and regulating their responsiveness to stimuli. Recently, to understand this balance through connectomics, a computational framework based on the Ising model was proposed. In Manos et al., the authors used a novel approach which involves a hybrid resting-state structural connectomes (rsSC). They used the Kuramoto model to produce signal to replicate both static and dynamic functional connectomes (FC), utilizing rsSC and traditional structural connectomes (SC) as coupling weight coefficients. Remarkably, the simulated FC with rsSC closely resembled observed FC data, surpassing simulations derived from SC.

In Maslennikov et al., the authors focused on the training of recurrent spiking neural networks aimed at generating spatiotemporal patterns represented as closed two-dimensional trajectories. Through analysis of spike trains within these networks, they assessed their dissimilarity utilizing the Victor–Purpura distance metric. Employing algebraic topology techniques, they manipulated matrices derived from the ranked entries of these distance matrices, focusing particularly on computing persistence barcodes and Betti curves. By juxtaposing the characteristics of various output patterns, they managed to reveal intricate associations between low-dimensional target signals and the multidimensional spike trains lying beneath, thus shedding light on the complex interplay within neural network dynamics.

Mean-field models have been instrumental in capturing essential aspects of epileptic seizure dynamics. Yet, the intricate mechanisms underlying seizure onset and propagation, particularly the involvement of specific brain areas, remain elusive. In Courson et al., the authors utilized computational techniques within The Virtual Brain framework and the Epileptor model to probe the relationship between the location and connectivity of an Epileptogenic Zone (EZ) in the mouse brain and the occurrence of focal seizures. They devised computational strategies to contain seizures, simulating medical interventions such as tissue resection or the application of anti-seizure drugs or neurostimulation. By selectively blocking specific connections informed by structural connectome data and graph network measurements or locally reducing outgoing connection weights from EZ areas, they demonstrated the ability to confine seizures around the EZ region. Their findings successfully identify the minimal connections required to prevent widespread seizures, prioritizing minimal surgical or medical intervention while preserving original structural connectivity and optimizing brain function.

## Author contributions

TM: Writing—original draft, Writing—review & editing. CA: Writing—review & editing. AB: Writing—review & editing. KI: Writing—review & editing.
